# Enhanced nuclear protein export in premature aging and rescue of the progeria phenotype by modulation of CRM1 activity

**DOI:** 10.1111/acel.13002

**Published:** 2019-07-15

**Authors:** Ian García‐Aguirre, Alma Alamillo‐Iniesta, Ruth Rodríguez‐Pérez, Griselda Vélez‐Aguilera, Elianeth Amaro‐Encarnación, Elizabeth Jiménez‐Gutiérrez, Alejandra Vásquez‐Limeta, Marco Samuel Laredo‐Cisneros, Sara L. Morales‐Lázaro, Reynaldo Tiburcio‐Félix, Arturo Ortega, Jonathan J Magaña, Steve J. Winder, Bulmaro Cisneros

**Affiliations:** ^1^ Department of Genetics and Molecular Biology, Center of Research and Advanced Studies (CINVESTAV‐IPN) Mexico City Mexico; ^2^ Laboratory of Protein Dynamics and Signaling, Center for Cancer Research‐Frederick National Cancer Institute, National Institutes of Health Frederick MD USA; ^3^ Department of Biomedical Science University of Sheffield Sheffield UK; ^4^ Department of Cognitive Neuroscience, Institute of Cellular Physiology National Autonomous University of Mexico (UNAM) Mexico City Mexico; ^5^ Department of Toxicology, Center of Research and Advanced Studies (CINVESTAV‐IPN) Mexico City Mexico; ^6^ Laboratory of Genomic Medicine, Department of Genetics National Rehabilitation Institute, “Luis Guillermo Ibarra Ibarra” Mexico City Mexico

**Keywords:** aging, cellular senescence, exportin CRM1, Hutchinson‐Gilford progeria syndrome, lamin B1, progerin

## Abstract

The study of Hutchinson–Gilford progeria syndrome (HGPS) has provided important clues to decipher mechanisms underlying aging. Progerin, a mutant lamin A, disrupts nuclear envelope structure/function, with further impairment of multiple processes that culminate in senescence. Here, we demonstrate that the nuclear protein export pathway is exacerbated in HGPS, due to progerin‐driven overexpression of CRM1, thereby disturbing nucleocytoplasmic partitioning of CRM1‐target proteins. Enhanced nuclear export is central in HGPS, since pharmacological inhibition of CRM1 alleviates all aging hallmarks analyzed, including senescent cellular morphology, lamin B1 downregulation, loss of heterochromatin, nuclear morphology defects, and expanded nucleoli. Exogenous overexpression of CRM1 on the other hand recapitulates the HGPS cellular phenotype in normal fibroblasts. CRM1 levels/activity increases with age in fibroblasts from healthy donors, indicating that altered nuclear export is a common hallmark of pathological and physiological aging. Collectively, our findings provide novel insights into HGPS pathophysiology, identifying CRM1 as potential therapeutic target in HGPS.

## 1 INTRODUCTION

Aging is a universal process that occurs across all living species; in humans, aging is characterized by a gradual decline in physical and psychological capacities that in turn cause morphological, metabolic, and cognitive alterations that limit the quality of life and ultimately provoke death. Premature aging syndromes, a group of extremely rare but devastating disorders that mimic physiological aging, have been proposed as models to decipher the molecular mechanisms underlying aging (Kubben & Misteli, 2017).

Hutchinson–Gilford progeria syndrome (HGPS), the most well‐studied progeroid syndrome, is typically caused by a silent point mutation (1824C > T) that activates a cryptic splicing site within exon 11 of the *LMNA* gene, resulting in the synthesis of a prelamin A mRNA that contains an internal deletion of 150 base pairs. Consequently, this leads to the translation of a mutant lamin A termed progerin, which harbors a deletion of 50 amino acids within its C‐terminus. Progerin is permanently farnesylated because the deletion eliminates the cleavage site for Zmpste24, the enzyme that removes the C‐terminal farnesyl group from prelamin A to render mature lamin A (Eriksson et al., 2003; De Sandre‐Giovannoli et al., 2003).

Progerin acts in a dominant gain‐of‐function manner by anchoring to the nuclear envelope and perturbing nuclear lamin function, thereby inducing a plethora of cellular defects, including aberrant nuclear morphology, altered DNA repair and gene expression, and telomere instability (Gonzalo, Kreienkamp, & Askjaer, 2017). The complex network of downstream effects by which progerin exerts its toxicity is far from understood; it is thought that progerin impacts on central cellular process, which in turn triggers a cascade of noxious downstream effects that ultimately cause aging.

Owing to the deleterious effect exerted by progerin on nuclear envelope, we hypothesized that the nucleocytoplasmic transport of proteins through the nuclear pore complex (NPC) (Christie et al., 2016) could be a primary target of progerin. Supporting our hypothesis, perturbation in the Ran GTPase gradient affecting nuclear import of specific proteins, namely Ubc9, and nucleoporin TPR was previously reported in HGPS cells (Cobb et al., 2016; Snow, Dar, Dutta, Kehlenbach, & Paschal, 2013). Furthermore, the nonclassic transport pathway mediated by transportin‐1 (TNPO1) is impaired in HGPS cells, due to cytoplasmic sequestration of TNPO1 by the microtubule network (Larrieu et al., 2018).

In this study, we aimed to analyze whether nuclear protein export is impaired in HGPS. Exportin‐1 (XPO1), also known as chromosomal region maintenance 1 (CRM1), binds to cargo proteins through a leucine‐rich nuclear export signal (NES) and transports the proteins across the NPC via a Ran‐GTP gradient (Ishizawa, Kojima, Hail, Tabe, & Andreeff, 2015; Kirli et al., 2015). We show for the first time that the CRM1‐driven nuclear protein export mechanism is abnormally enhanced in HGPS fibroblasts and that this novel hallmark is critically involved in the disease, because pharmacological inhibition of CRM1 rescues the HGPS cellular phenotype and exogenous overexpression of CRM1 recapitulates HGPS in normal fibroblasts.

## 2 RESULTS

### 2.1 Enhanced CRM1‐driven nuclear protein export is a novel hallmark of HGPS cells

**Figure 1:**
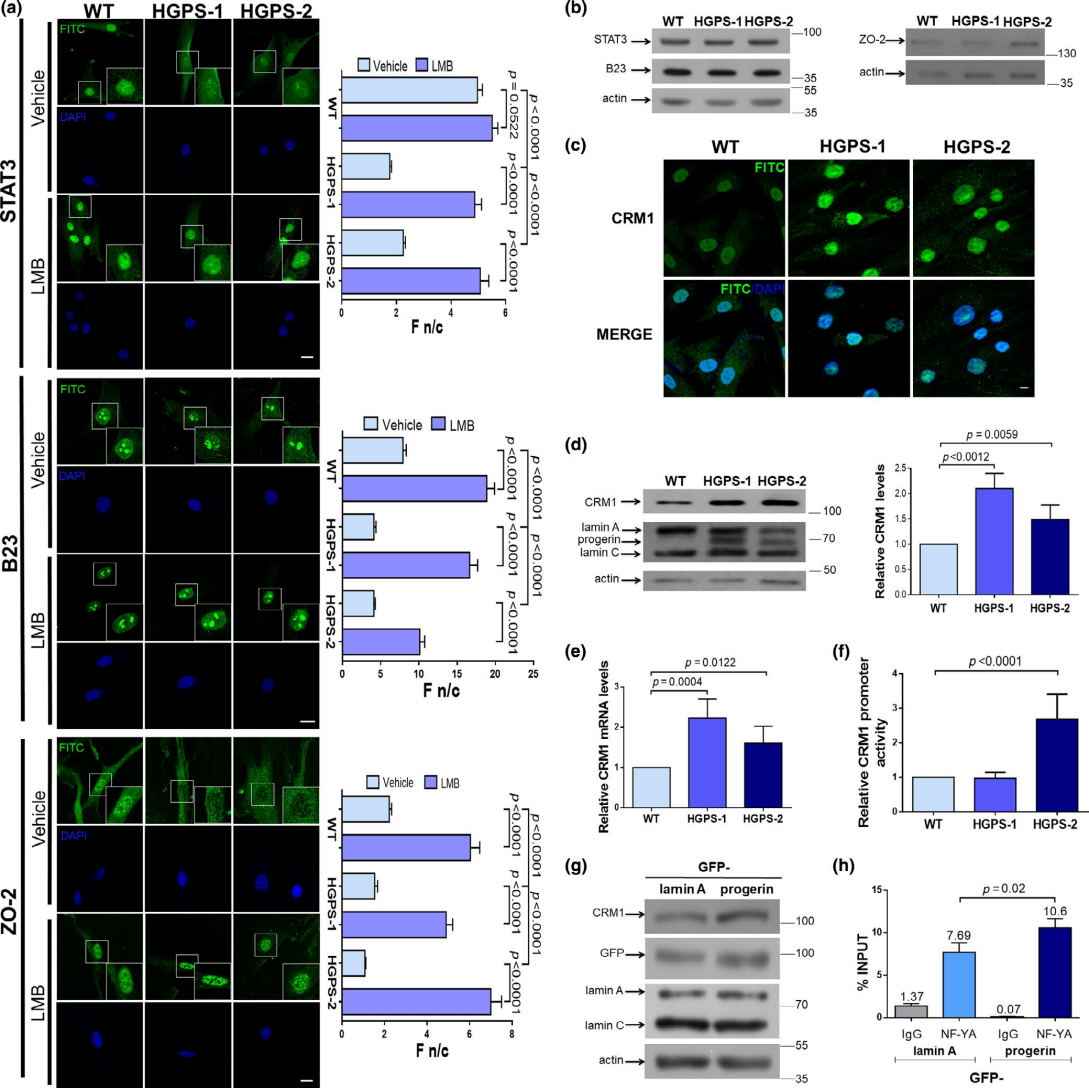
Mislocalization of NES‐containing proteins is caused by progerin‐mediated overexpression of CRM1. (a) Wild‐type (WT) and HGPS cells, treated for 24 hr with 10 nM LMB or vehicle alone, were immunolabeled for the indicated proteins (bar, 10 µm). The Fn/c ratio was calculated (*n* = 50 cells), and significant differences were determined by Mann–Whitney U test (graphs). (b) Protein levels of STAT3, B23, ZO‐2, and actin (loading control) were analyzed, and typical gels from two independent experiments are shown. (c) WT and HGPS cells were immunolabeled for CRM1 and analyzed by CLSM. Typical images from 3 independent assays are shown (bar 10 µm). (d) Lysates from WT and HGPS cells were subjected to Western blot using antibodies against CRM1, lamin A/C, or actin (loading control), and relative CRM1 levels were obtained (graph). (e) CRM1 messenger RNA expression was examined by qRT‐PCR. (d‐e) Significant differences were determined by unpaired *t *test. (f) WT and HGPS cells were transfected with both Luciferase reporter construct containing the human CRM1 promoter and Renilla luciferase vector, which was used to normalize transfection efficiency. Enzymatic activities were estimated after incubation for 48 hr as described in Methods. Data represent mean ± SEM of three independent experiments (unpaired *t* test). (g) Lysates from HeLa cells stably transfected to express GFP‐lamin A or GFP‐progerin were subjected to Western blotting using antibodies against CRM1, GFP, or actin (loading control). (h) HeLa cells expressing GFP‐lamin A or GFP‐progerin were subjected to chromatin immunoprecipitation (ChIP) with anti‐NF‐YA antibodies followed by PCR for the CRM1 promoter region. Data correspond to two independent experiments in triplicate

To ascertain whether the CRM1‐mediated nuclear protein export mechanism is impaired in HGPS, we evaluated the subcellular distribution of six different proteins whose nuclear export depends on NES recognition by CRM1 (STAT3, B23, ZO‐2, cyclin B1, Dp71, and HDAC1). Two different HGPS fibroblast cultures (HGPS‐1 and HGPS‐2) and a passage‐matched fibroblast control culture (WT) were examined using confocal laser scanning microscopy (CLSM). There was a significant decrease in the nuclear localization with a concomitant increase in the cytoplasmic labeling for all the analyzed proteins in both HGPS cell cultures, with the exception of HDAC1 that was found to be mislocalized only in HGPS‐2 cells (Figure 1a and Figure S1). Quantitative analysis of the fluorescence nuclear/cytoplasmic ratio (Fn/c) corroborated these observations (see graphs). Nuclear accumulation of NES‐containing proteins was restored to a similar level to that of WT cells upon treatment of HGPS cells for 1 day with 1 nM leptomycin B (LMB), a specific CRM1 inhibitor (Figure 1a and Figure S1). This clearly indicates that their nuclear depletion is caused by enhanced nuclear export activity rather than nuclear import failure. Next, we analyzed the expression of NES‐containing proteins in HGPS cells. The levels of STAT3 and B23 were similar between WT and HGPS cells, while an apparent increase for ZO‐2 was observed only in HGPS‐2 fibroblasts (Figure 1b).

### 2.2 Enhanced nuclear protein export in HGPS cells is caused by progerin‐induced CRM1 overexpression

We hypothesized that the exacerbated nuclear export activity of HGPS cells might be consequence of CRM1 overexpression. Consistent with this idea, elevated CRM1 protein levels were found in HGPS cells (Figure 1c,d), with qRT‐PCR experiments showing a corresponding increase in CRM1 mRNA levels (Figure 1e). Given that CRM1 overexpression is primarily regulated at the transcriptional level, we next analyzed CRM1 promoter activity in HGPS cells by promoter reporter assays. An increase in CRM1 promoter activity was found in HGPS‐2 but not HGPS‐1 cells, compared with WT cells (Figure 1f).

To ascertain whether CRM1 overexpression is causally linked to progerin, we evaluated whether exogenous expression of progerin is sufficient to elicit increased CRM1 levels in HeLa cells. Expression of GFP‐progerin but not GFP‐lamin A resulted in nuclear morphology aberrations (Figure S2a), decreased H3K9m3 levels (Figure S2b), and increased CRM1 levels (Figure 1g); the latter appears to provoke in turn cytoplasmic mislocalization of the NES‐containing STAT3 (Figure S2c). We then evaluated whether exogenously expressed progerin induces CRM1 promoter activity, using ChIP‐qPCR assays to assess binding activity of NF‐YA, a transcription factor that positively regulates the CRM1 promoter (van der Watt & Leaner, 2011). The binding activity of NF‐YA to the CRM1 promoter region was found to be augmented by ~30% in GFP‐progerin‐expressing cells, compared with those expressing GFP‐lamin A (Figure 1h).

Because progerin toxicity is attributed at least in part to its farnesyl moiety, we next examined whether inhibition of progerin farnesylation using the farnesyltransferase inhibitor (FTI) lonafarnib would reduce overexpression of CRM1 in HGPS cells. As previously reported (Noda et al., 2015), treatment with 25 µM of lonafarnib for 3 days elicited a significant decrease in both nuclear blebbing (Figure S3a) and progerin levels (Figure S3b). Supporting our idea, the decrease in progerin levels resulted in CRM1 downregulation (Figure S3b).

### 2.3 Pharmacological attenuation of CRM1 activity rescues premature senescence in HGPS cells by improving lamin B1 levels

**Figure 2:**
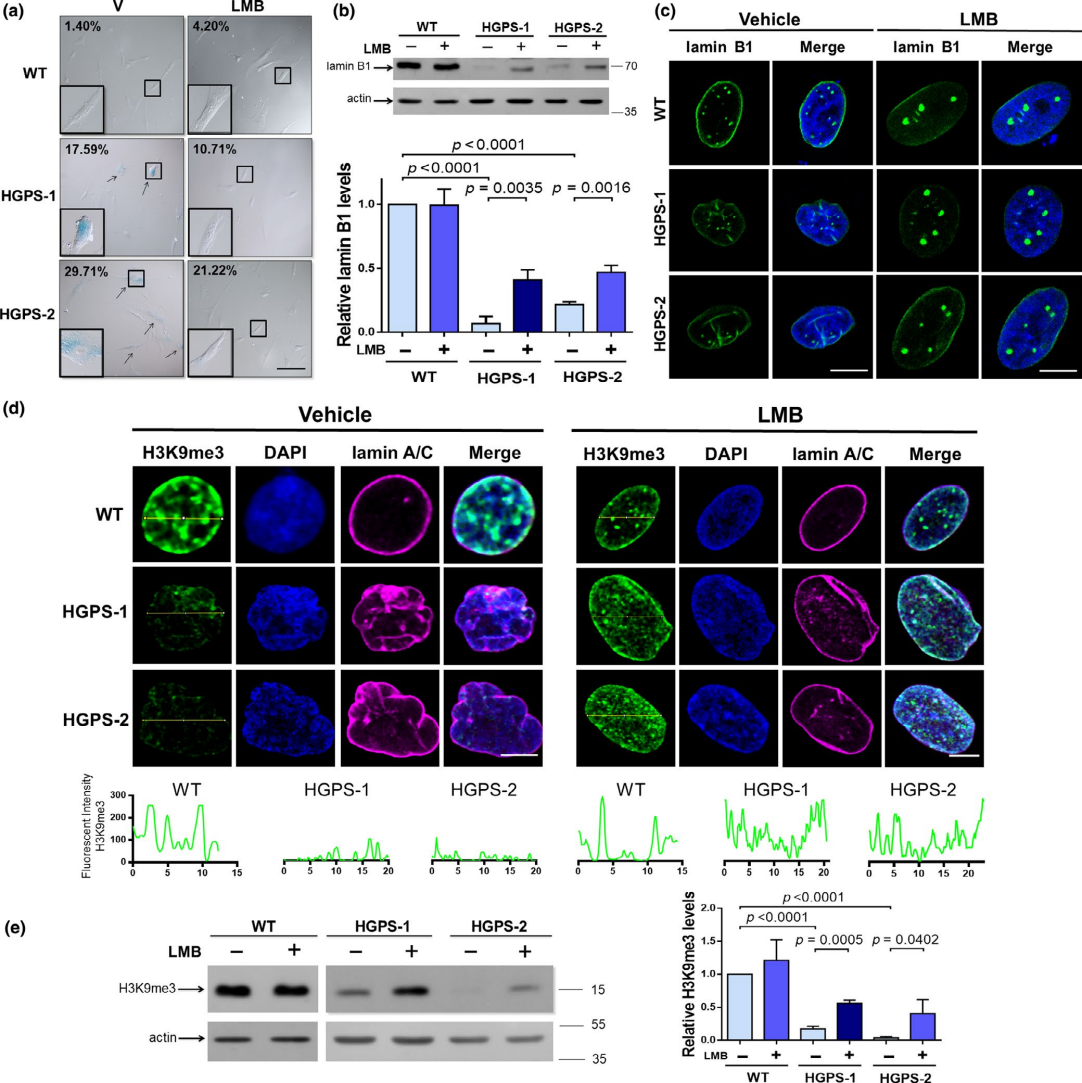
LMB‐mediated inhibition of CRM1 prevents cellular senescence by improving lamin B1 levels. (a) WT and HGPS fibroblasts were treated for 2 days with 1 nM LMB or vehicle alone. The activity of β‐galactosidase was assessed, and representative images are shown. Bar = 100 µM. The percentage of senescent cells was calculated from 3 separate experiments (*n* = 300 cells for each condition). (b) Lysates from WT and HGPS fibroblasts, previously treated for 6 days with 50 nM LMB or vehicle, were analyzed by Western blotting using antibodies against lamin B1 and actin (control). Lamin B1 levels were assessed (bottom panel). (c) WT and HGPS fibroblasts treated with 50 nM LMB or vehicle alone for 3 days were immunostaining for lamin B1, and typical images are shown. Bar, 10 µm. (d) WT and HGPS fibroblasts treated with 50 nM LMB or vehicle for 6 days were analyzed by CLSM, using antibodies against H3K9me3 and lamin A/C. Representative images are shown. B Bar, 10 µm. *Bottom.* Line profile analysis showing H3K9me3 fluorescence pattern. (e) Lysates from HGPS cells treated as peer C were analyzed by Western blotting using antibodies against H3K9me3 and actin (control). Relative H3K9me3 levels are shown (right graph). (b and e) Significant differences were determined by unpaired *t *test

We wondered whether enhanced CRM1 activity might be part of the molecular basis underlying HGPS. To address this, we evaluated whether amelioration of CRM1 activity through LMB treatment has an impact on cellular senescence. Remarkably, LMB treatment for 2 days resulted in a significant reduction of senescence cells in HGPS cultures, as determined by senescence‐associated β‐galactosidase (SA‐β‐gal) staining (Figure 2a). Consistent with this, the percentage of HGPS‐1 and HGPS‐2 cells arrested at G0/G1 phase of the cell cycle decreased upon treatment with LMB, as revealed by flow cytometry analysis (Figure S4a). We next analyzed the effect of LMB on cell proliferation. Treatment with LMB for 3 days resulted in significantly decreased cell growth in both WT and HGPS cells; however, it provoked only a slight increase in cell death by the third day, as shown by MTT and annexin V assays (Figure S4b,c, respectively). Since decreased lamin B1 expression is associated with senescence (Freund, Laberge, Demaria, & Campisi, 2012; Shimi et al., 2011), we were prompted to analyze whether treatment with LMB has a positive effect on lamin B1 levels. A ~80% decrease in lamin B1 levels was observed in HGPS cells incubated with vehicle alone. Nevertheless, partial restitution of lamin B1 expression was obtained after incubation of HGPS fibroblasts with LMB (50 nM) for 6 days, while no changes were found in WT cells upon treatment (Figure 2b). Redistribution of lamin B1 into distinct nucleoplasmic foci was observed in LMB‐treated HGPS cells (Figure 2c). It is thought that nucleoplasmic foci of lamin B1 correspond to nascent lamin B1 incorporated in the nucleoplasmic reticulum in interphase nuclei (Drozdz, Jiang, Pytowski, Grovenor, & Vaux, 2017).

We considered that restoration of lamin B1 levels might consequently reduce those HGPS characteristics governed by lamin B1 (Camps et al., 2014). We then examined the impact of LMB treatment on heterochromatin organization using the heterochromatin marker H3K9me3. Supporting our idea, recovery of both nuclear staining and protein levels of H3K9me3 was evidenced in HGPS cells after LMB treatment (Figure 2d,e, respectively).

**Figure 3:**
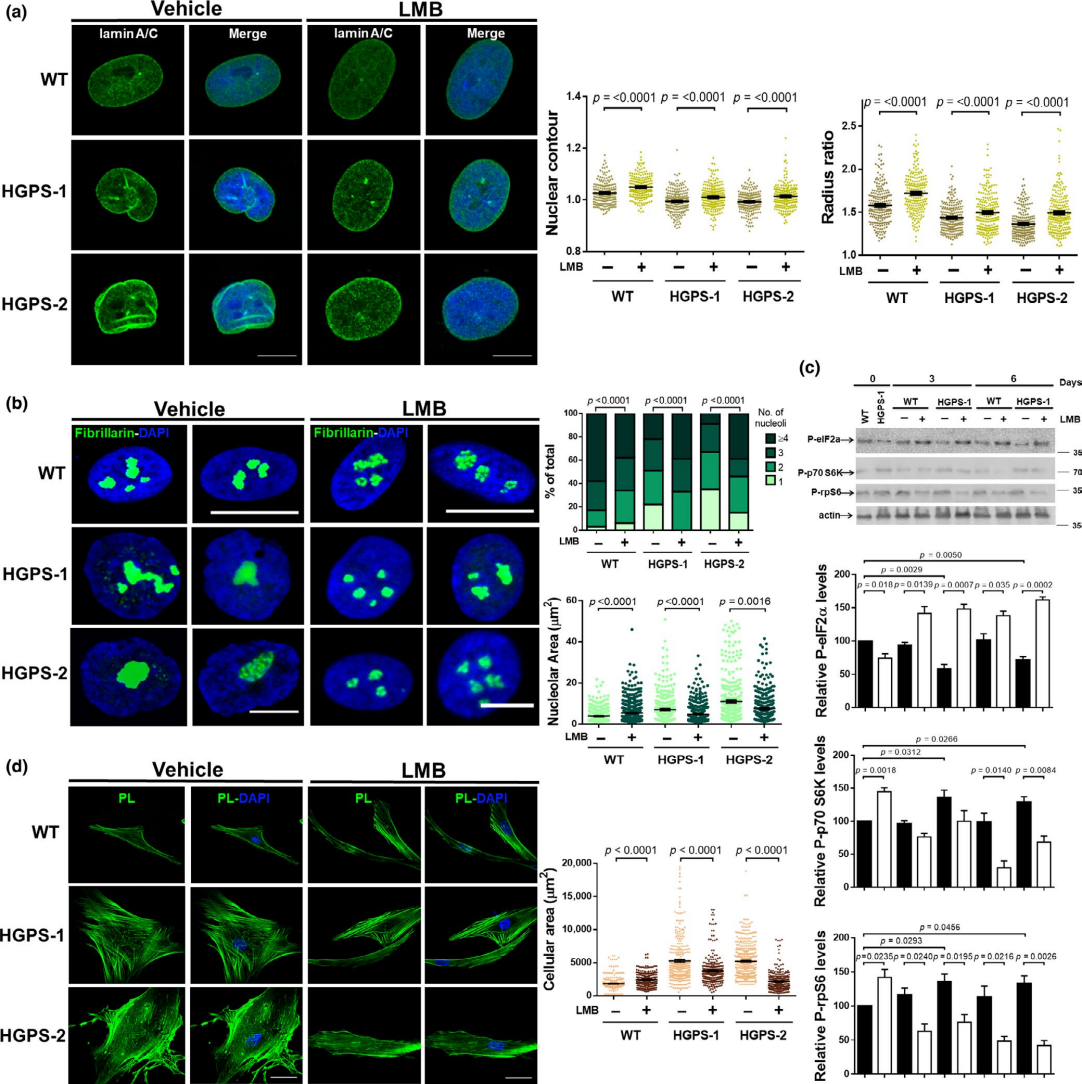
LMB‐mediated inhibition of CRM1 alleviates aging features of HGPS cells. (a) WT and HGPS fibroblasts were treated for 3 days with 50 nM LMB or vehicle alone. Cells were immunolabeled for lamin A/C before being analyzed by CLSM, and representative images are shown. Bar, 10 µm. *Right.* Nuclear morphometric analysis was carried out as described in Methods (*n* = 200 nuclei for each condition), with significant differences determined by Mann–Whitney U test. (b) WT and HGPS fibroblasts, treated as peer A, were immunostained for fibrillarin to decorate nucleoli. Bar, 10 µm. *Right.* Nucleolar area was determined as described in Methods (*n* > 600 nucleoli per condition). Number of nucleoli per cell was estimated (bottom panel; *n* > 80 cells per sample), and significant differences were determined by Mann–Whitney U test. (c) Lysates from WT and HGPS‐1 fibroblasts, previously treated for 0, 3, or 6 days with 1 nM LMB or vehicle, were analyzed by Western blotting using antibodies against the indicated proteins. Relative protein levels from three independent experiments are shown (bottom graphs), with significant differences determined by unpaired *t *test. (d) WT and HGPS fibroblasts were treated as peer A. Cells were labeled with phalloidin to visualize actin cytoskeleton. Bar, 50 µM. *Right.* The cellular area was estimated (*n* > 300 cells), with significant differences determined by Mann–Whitney U test

We next examined the effect of LMB on nuclear morphology, using nuclear morphometric analyses. Nuclear defects were commonly found in vehicle‐treated HGPS fibroblasts immunostained for lamin A/C, compared with WT cells (Figure 3a and graphs). Remarkably, ovoid‐shaped nuclei devoid of irregularities that resemble WT nuclei were evident after treatment of HGPS cells for 3 days with LMB (Figure 3a). Nuclear morphometric analyses confirmed significant differences between vehicle‐ and LMB‐treated HGPS cells (right graphs). Collectively, these data indicate that mitigation of CRM1 activity through LMB treatment delayed the progression of HGPS cells to senescence by preventing lamin B1 downregulation.

### 2.4 Pharmacological modulation of CRM1 activity ameliorates aging features of HGPS cells

To analyze in depth the effect of LMB‐mediated CRM1 inhibition on senescence, two distinctive features of senescent cells, namely enlarged nucleolar morphology and senescent cellular morphology, were examined in HGPS fibroblasts. As reported previously, (Buchwalter & Hetzer, 2017), a single or two prominent nucleoli with increased nucleolar area were observed in most HGPS cells (Figure 3b). Notably, administration of LMB to HGPS cultures for 3 days resulted in increased number of nucleoli per cell (Figure 3b), with smaller nucleolar area (right graphs), which were comparable in size to wild‐type nucleoli. Previously, the expansion of nucleoli was linked to elevated protein synthesis in HGPS cells (Buchwalter & Hetzer, 2017). Therefore, we were prompted to analyze whether alleviation of nucleolar expansion by LMB treatment has an effect on global protein synthesis by monitoring the phosphorylation state of the α subunit of eukaryotic initiation factor 2 (eIF2α), the p70 ribosomal protein S6 kinase (p70 S6K) and its substrate, and the ribosomal protein S6 (rpS6). Consistent with elevated protein synthesis, HGPS‐1 cells treated with vehicle alone showed decreased p‐eIF2a levels but elevated levels of p‐p70 S6K and p‐rpS6K (Figure 3c). By contrast, LMB treatment for 3 and 6 days resulted in increased p‐eIF2α levels but reduced phosphorylated levels of both p70 S6K and rpS6 in HGPS‐1 cells (Figure 3c), which is consistent with a translational repression effect mediated by LMB. With respect to the senescent cell morphology, LMB‐mediated inhibition of CRM1 activity shifted the flattened and expanded morphology of HGPS fibroblasts to the typical fusiform morphology of WT fibroblasts (Figure 3d), with a concomitant decrease in cell size (right graph).

Finally, we assessed whether treatment with LMB has an effect on progerin expression. Interestingly, progerin levels were found to significantly decrease in HGPS‐2 cells but not HGPS‐1 cells upon LMB treatment (Figure S4c).

### 2.5 Ectopic overexpression of CRM1 recreates the HGPS phenotype in normal human fibroblasts

**Figure 4:**
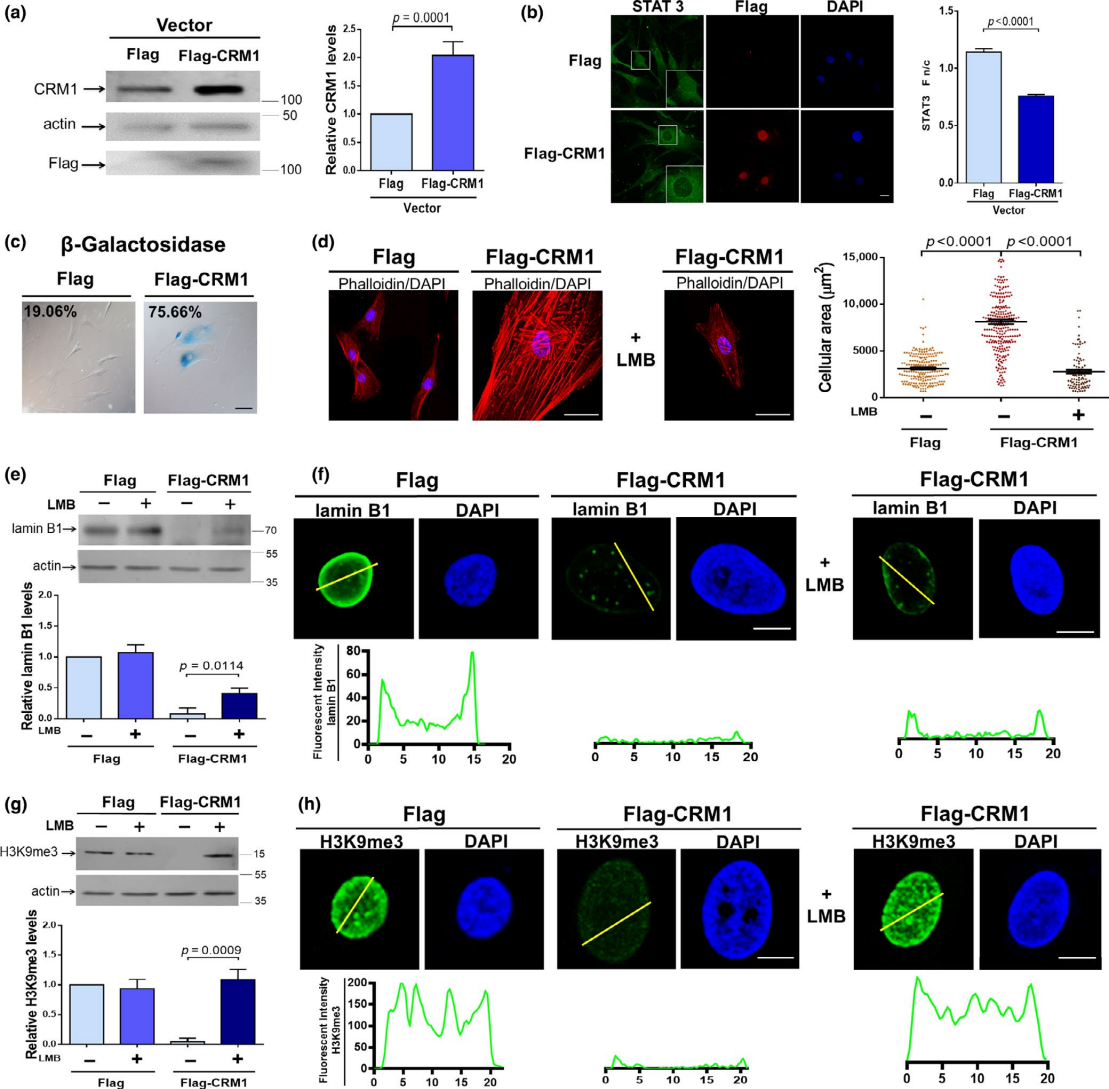
Ectopic overexpression of CRM1 induces premature aging phenotype in normal primary fibroblasts. Human primary fibroblasts derived from a healthy donor were stably transfected to express Flag‐CRM1 or Flag alone. (a) Lysates were subjected to Western blotting using antibodies against CRM1, Flag, or actin (loading control). Relative CRM1 levels are shown (right panel). (b) Subcellular distribution of STAT3 was evaluated by CLSM and its n/c ratio estimated (graph). Bar, 10 µM. (c) The activity of β‐galactosidase was measured, and the percentage of senescent cells was estimated from 3 independent experiments (*n* = 300 cells for condition). Bar, 100 µM. (a‐c) Significant differences were determined by unpaired *t *test. (d) Transfected fibroblasts were labeled with phalloidin to visualize actin. *Right*. Flag‐CRM1‐expressing fibroblasts were treated with 50 nM LMB or vehicle alone for 3 days, prior to being stained with phalloidin and analyzed by CLSM. Bar, 50 µM. The cellular area was determined (*n* > 150 cells for condition), and significant differences were determined by Mann–Whitney U test. (e) Lysates from fibroblasts expressing Flag or Flag‐CRM1, which were previously treated with 50 nM LMB or vehicle alone for six days, were analyzed by Western blotting with antibodies against lamin B1 and actin (control). *Bottom*. Relative lamin B1 levels are shown. Significant differences were determined by unpaired *t *test. (f) Transfected fibroblasts with or without LMB treatment were immunostained for lamin B1, and typical images are shown. Bar, 10 µm. *Bottom.* Line profile analysis showing lamin B1 fluorescence pattern. (g) WT fibroblasts stably expressing Flag or Flag‐CRM1 were treated as peer (e) prior to be analyzed by Western blotting for H3K9me3. *Bottom.* Relative H3K9me3 levels are shown, and significant differences were determined by unpaired *t *test. (h) Nuclear distribution of H3K9me3 was analyzed in the indicated transfected fibroblasts with or without LMB treatment. *Bottom*. Line profile analysis showing the H3K9me3 fluorescence pattern

Next, we ascertained whether exogenous overexpression of CRM1 is sufficient to mimic progeroid hallmarks in normal fibroblasts. A ~100% increase in CRM1 levels was observed after stable transfection of WT fibroblasts with a vector expressing FLAG‐CRM1, compared to WT cells expressing FLAG alone (Figure 4a). Cytoplasmic mislocalization of the NES‐containing protein STAT3 confirmed accelerated nuclear export activity in cells overexpressing CRM1 (Figure 4b). Remarkably, most of CRM1‐overexpressing WT fibroblasts became senescent (75%), as shown by SA‐β‐gal staining and the acquisition of a flattened and extended cellular morphology (Figure 4c,d, respectively). Furthermore, forced overexpression of CRM1 in WT fibroblasts resulted in decreased lamin B1 levels (Figure 4e–f), with the consequent loss of H3k9m3 (Figure 4g–h). Interestingly, pharmacological inhibition of CRM1 in FLAG‐CRM1‐expressing fibroblasts alleviated the senescent morphology (Figure 4d) and prevented both lamin B1 downregulation (Figure 4e,f) and the loss of heterochromatin (Figure 4g,h).

**Figure 5:**
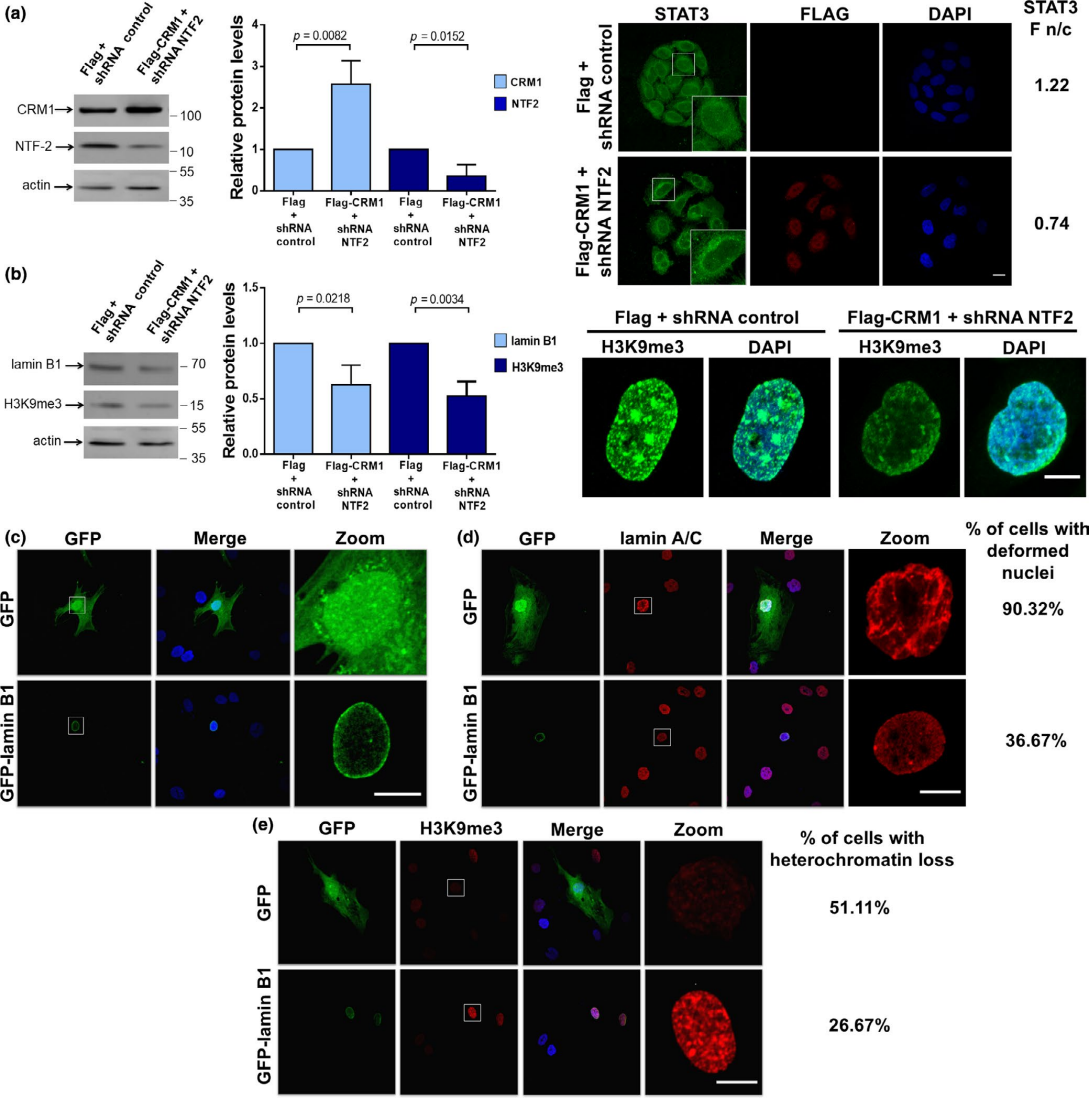
(a‐b) Enhanced nuclear export activity due to CRM1 overexpression overcomes deficient Ran gradient in HeLa cells. Cells were double‐transfected to stably expressed Flag‐CRM1 or Flag alone, and a shRNA against NTF2 gene or a shRNA control. (a) Lysates from the transfected cells were analyzed by Western blotting using antibodies against CRM1, NTF2, and actin (control). *Middle.* Relative protein levels were assessed from three independent experiments (unpaired *t *test). *Right*. Distribution of STAT3 was analyzed in the indicated transfected cells. Bar, 20 µM. (b) Transfected cell lysates were analyzed by Western blotting with antibodies against lamin B1, H3K9me, and actin (control). *Middle.* Data correspond to 3 independent experiments (unpaired *t *test). *Right*. Distribution of H3K9me3 was analyzed in the indicated transfected cells. Bar, 20 µM. (c–e) Restoration of lamin B1 expression in HGPS cells (c) HGPS‐1 cells were transiently transfected to express GFP‐lamin B1 or GFP alone. Transfected cells were immunolabeled for lamin A/C (d) and H3K9m3 (e) to estimate the percentage of cells with aberrant nuclear morphology and heterochromatin loss, respectively. Bar, 10 µM

The gradient of Ran, a key regulator of nucleocytoplasmic transport, was previously reported as disrupted in HGPS cells (Datta, Snow, & Paschal, 2014). We therefore investigated whether enhanced nuclear export activity via CRM1 overexpression can occur in cells with a deficient Ran gradient. Since depletion of the Ran import factor NTF2 (nuclear transport factor 2) is sufficient to disrupt the Ran gradient (Datta et al., 2014; Dworak et al., 2019), HeLa cells were double‐transfected to express CRM1 and a shRNA against NTF2. Interestingly, stably transfected HeLa cells having both overexpressed levels of CRM1 and depleted levels of NTF2 (Figure 5a) still showed elevated nuclear export activity, as shown by cytoplasmic mislocalization of the NES‐containing protein STAT3 (right panel). Consequently, these cells acquired different aging marks, including depleted levels of lamin B1 and H3K9m3 (Figure 5b). Collectively, these data imply that enhancing CRM1 activity is necessary and sufficient to induce the cells to age prematurely.

### 2.6 Exogenous restoration of lamin B1 levels partially alleviates the HGPS phenotype

To ascertain whether improvement of lamin B1 levels by LMB treatment is the basis of the HGPS phenotype rescue, the expression of lamin B1 was exogenously restored in HGPS‐1 cells by stably transfecting a vector expressing GFP‐lamin B1. GFP‐lamin B1 was properly targeted to the nuclear envelope, while GFP alone was localized throughout all the cells (Figure 5c). Remarkably, HGPS cells expressing GFP‐lamin B1 showed improved nuclear morphology (Figure 5d) and recovery of peripheral heterochromatin (Figure 5e), compared with HGPS‐1 cells expressing GFP alone. However, restoration of lamin B1 expression failed to alleviate the senescent morphology (data not shown).

### 2.7 The expression and activity of CRM1 increase during normal aging

**Figure 6:**
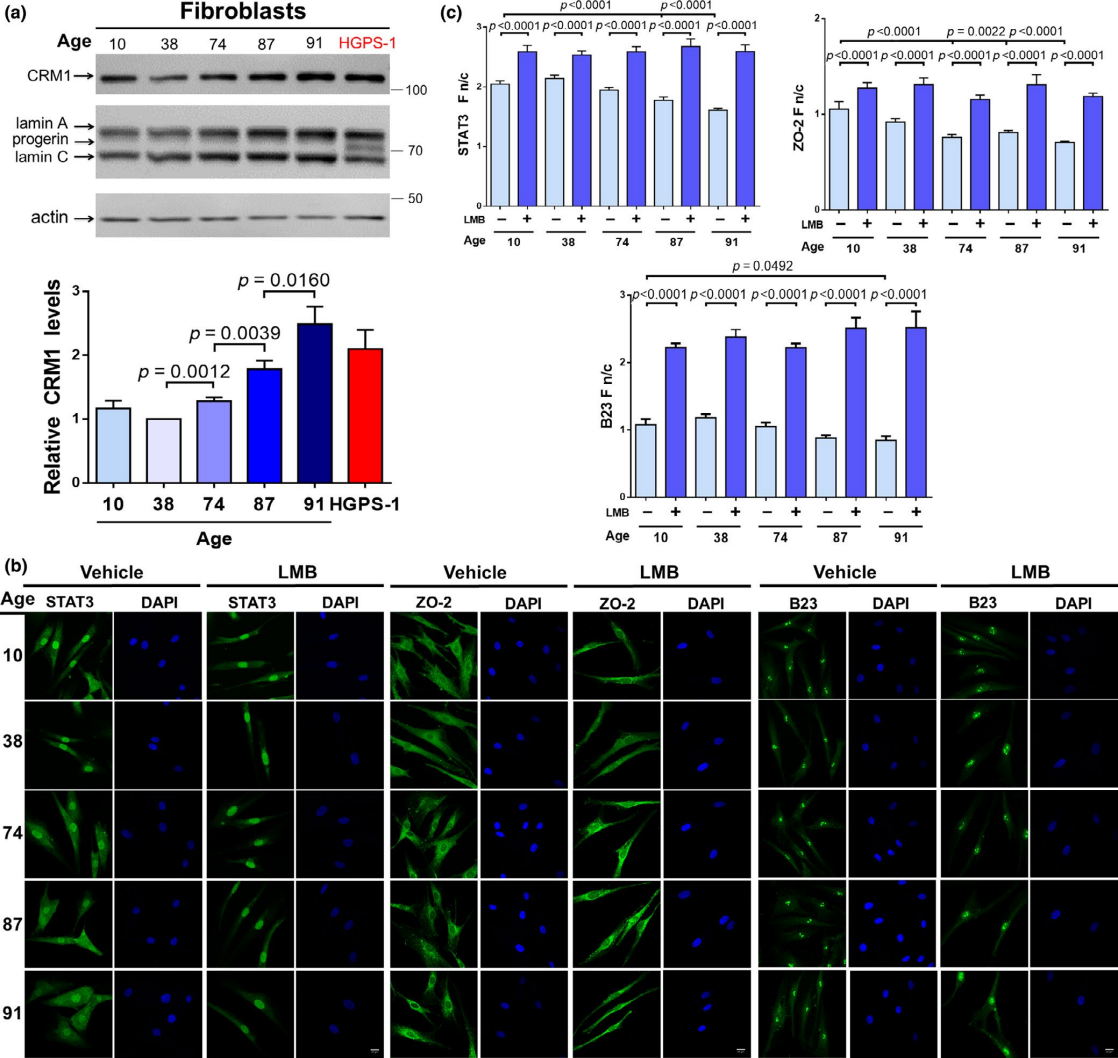
CRM1 expression and activity increased during normal aging. (a) Primary human fibroblast from healthy individuals of varying ages or HGPS‐1 fibroblasts were analyzed by Western blotting using antibodies against CRM1, lamin A/C, and actin (control). CRM1 expression is shown (bottom panel; unpaired *t *test). (b) Localization of the NES‐containing proteins STAT3, Z0‐2, and B23 was evaluated in the indicated fibroblast cultures, treated with LMB or vehicle alone for 24 hr. Typical images are shown. Bar, 20 µM. (c) The n/c ratio of STAT3, Z0‐2, and B23 was calculated as peer Methods (*n* = 50 cells; Mann–Whitney U test)

We hypothesized that the CRM1‐mediated nuclear export system could be dysregulated during normal aging as well. Thus, we analyzed expression and activity of CRM1 in a panel of primary human fibroblasts from healthy individuals ranging from 10 to 91 years of age. Interestingly, we found a significant and direct correlation between aging and increased CRM1 levels in individuals between 38 and 91 years of age (Figure 6a). Cytoplasmic mislocalization of the NES‐containing proteins STAT3, Z0‐2, and B23 was evident in fibroblasts from 74‐ to 91‐year‐old individuals (Figure 6b), with Fn/C analysis corroborating these observations (Figure 6c); further treatment with LMB restored predominant nuclear localization of NES‐containing proteins, similar to that observed in fibroblasts from 10‐ to 38‐year‐old individuals (Figure 6b‐c). Altogether, these data suggest that enhanced CRM1 activity is a hallmark of physiological aging.

## 3 DISCUSSION

In this study, we show for the first time that the CRM1‐driven nuclear protein export mechanism is abnormally enhanced in HGPS cells due to overexpression of CRM1. We revealed that upregulation of CRM1 in HGPS cells is controlled at the transcriptional level through induction of CRM1 promoter activity, at least for HGPS‐2 cells. Furthermore, a causal link between progerin and CRM1 overexpression was demonstrated because ectopic expression of progerin in HeLa cells elicited elevated CRM1 levels, at least in part through enhancing the binding activity of NF‐YA (a positive transcription factor) to the CRM1 promoter region, and because treatment of HGPS cells with FTI that decreased progerin levels resulted in turn in downregulation of CRM1. We speculated that exacerbated nuclear export activity might be part of the molecular basis underlying HGPS. As proof of concept, we evaluated whether inhibition of CRM1 activity by pharmacological treatment with LMB has a therapeutic effect on the HGPS phenotype. Remarkably, we found alleviation of virtually all HGPS hallmarks upon treatment with LMB, including reduced number of SA‐β‐gal positive cells, recovery of peripheral heterochromatin, and rescue of lamin B1 downregulation, nucleolar expansion, augmented protein synthesis, and the senescent cellular morphology. Furthermore, ectopic overexpression of CRM1 enabled normal fibroblasts to acquire all the aging marks aforementioned. Thus, CRM1 overexpression is sufficient to recapitulate an aging phenotype in the absence of progerin, which indicates that a dysregulated nuclear protein export is an early key event in the pathway leading to HGPS.

Most of the cellular processes rescued by LMB treatment are intimately related to lamin B1, including nuclear morphology (Vergnes, Peterfy, Bergo, Young, & Reue, 2004), organization of peripheral heterochromatin, nucleolar plasticity (Camps et al., 2014), and cellular senescence (Dreesen, Ong, Chojnowski, & Colman, 2013; Freund et al., 2012; Lukasova, Kovarik, & Kozubek, 2018; Shimi et al., 2011). Therefore, it is likely that rescue of the HGPS phenotype by treatment with LMB relies mainly on the improvement of lamin B1 levels. Consistent with this, exogenous expression of lamin B1 resulted in improved nuclear morphology and the recovery of peripheral heterochromatin, but failed to alleviate the senescent morphology of HGPS cells. Thus, alleviation of the HGPS aging marks appears to occur through more than one mechanism; further experiments are required to fully delineate pathways underlying the correction of aging marks in response to CRM1 inhibition.

A broad spectrum of treatments against HGPS has been tested in HGPS fibroblasts (Harhouri et al., 2018), and most of these are designed to target progerin. Blocking of progerin farnesylation by administration of farnesyltransferase inhibitors corrected nuclear structure but failed to alleviate other aging‐associated marks (Glynn & Glover, 2005; Toth et al., 2005)*,* while depletion of progeria via rapamycin‐mediated activation of autophagy improved nuclear morphology and delayed the onset of senescence (Cao et al., 2011). In a similar manner, proteasome inhibitor MG132 enhanced proliferation and decreased cellular senescence by promoting progerin degradation (Harhouri et al., 2017). Finally, treatment with the chemical JH4 ameliorated nuclear defects and prevented premature senescence by interfering with the harmful interaction between progerin and lamin A (Lee et al., 2016). Alternative strategies aimed to repair downstream effects of progerin have been proposed as well; treatment with methylene blue rescued not only nuclear morphology and heterochromatin loss but also mitochondrial function (Xiong et al., 2016), while administration of the ROCK inhibitor Y‐27632 ameliorated nuclear morphology defects and DNA double‐strand breaks along with decreased ROS levels and improvement of mitochondrial function (Kang et al., 2017). In this scenario, pharmacological modulation of CRM1 using selective inhibitors clearly provides a viable and promising therapy against HGPS, because this treatment specifically targets and corrects the nuclear export mechanism and, more importantly, because it alleviates the largest number of HGPS cellular characteristics (Graphical abstract), compared with the aforementioned treatments. Furthermore, synthetic selective inhibitors of CRM1 with pharmacological properties superior to LMB (selinexor/KPT‐330), which have shown to be well‐tolerated in human cancer clinical trials (Mahipal & Malafa, 2016), would facilitate future in vivo evaluation of this therapy in HGPS animal models.

Interestingly, we found increased CRM1 levels in primary fibroblast from healthy aged donors; however, no progerin was detected in these cell cultures. In analogy to the mechanism underlying CRM1 overexpression in cancer cells (van der Watt & Leaner, 2011), we hypothesized that upregulation of CRM1 in fibroblasts from aged individuals could be controlled by modulation of the binding activity of transcription factors involved in CRM1 promoter activity. Thus, enhanced CRM1‐mediated nuclear export activity appears to be a key converging mechanism in both pathological and physiological aging. Consistent with this notion, silencing or pharmacological inhibition of CRM1 resulted in extended lifespan in *C. elegans* (Silvestrini et al., 2018).

In conclusion, we reveal that enhanced nuclear protein export is a new hallmark of both HGPS and normal aging. Furthermore, we provide evidence that pharmacological inhibition of CRM1 activity alleviates the progeroid cellular phenotype (Graphical abstract), which delineates CRM1 as potential therapeutic target for HGPS and other aging‐related diseases.

## 4 EXPERIMENTAL PROCEDURES

### 4.1 Cell culture, treatments, and transfection

Primary human dermal fibroblasts from patients with HGPS and healthy donors (see Table 1) were cultured in MEM (Invitrogen) supplemented with 15% FBS (Invitrogen), nonessential amino acids, and antibiotics. All experiments were carried out with fibroblast cultures at passage numbers 12–16. Where indicated, fibroblasts were treated with 1 nM leptomycin B (LMB; Sigma‐Aldrich) for 1, 3, or 6 days diluted in ethanol to a final concentration of 0.1% in the culture medium, or with 25 µM of the farnesyl transferase inhibitor (FTI) lonafarnib (Sigma‐Aldrich) for three days. Fibroblasts were transfected with Lipofectamine 3,000 following manufacturer's protocol (Invitrogen). Stably transfected fibroblasts were obtained by culturing them for 12 days with 200 μg/ml G418 (Invitrogen). HeLa cells were double‐transfected with pFlag‐CRM1 vector and vector expressing either a short hairpin RNA (shRNA) specific for the human nuclear transport factor 2 gene (NUTF2) or a scrambled shRNA control (GeneCopoeia, Inc.), using Lipofectamine 2000 (Invitrogen). Stably transfected HeLa cells were obtained by culturing them for 6 days in the presence of 1 μg/ml puromycin and 800 μg/ml G418. Cloning strategies to obtain vectors pEGFP‐C1‐LB1, pFLAG‐CRM1, and pCRM1 promoter are provided under request.

**Table 1:** List of human dermal fibroblast cultures used in this study

Culture	Origin	LMNA mutation	Age of donor at biopsy	Gender
AG08469 (WT)	Coriell	None	38	M
AG11513 (HGPS−1)	Coriell	G608G	8	F
AG11498 (HGPS−2)	Coriell	G608G	14	M
AG08470	Coriell	None	10	F
AG06290	Coriell	None	74	M
AG10884	Coriell	None	87	M
AG07725	Coriell	None	91	M

### 4.2 Promoter reporter assays

WT, HGPS‐1, and HGPS‐2 fibroblasts were transfected as above with both the CRM1 promoter vector, which expresses Firefly luciferase under the control of human CRM1 promoter, and the Renilla luciferase vector as a control to normalize for differences in transfection efficiency. After 48 hr, the luciferase activity was measured using Dual Luciferase Assay kit (Promega).

### 4.3 Quantitative chromatin immunoprecipitation (Q‐ChIP) assays

Q‐ChIP assays were performed as reported previously (Morales‐Lazaro et al., 2010), using 7.8 µg of mouse anti‐NF‐YA antibodies or 7.8 µg of mouse IgG (irrelevant control). 20 ng of DNA was used to perform quantitative PCR (qPCR) using SYBR Green and primers specific to amplify the CRM1 promoter region (van der Watt & Leaner, 2011).

### 4.4 Indirect immunofluorescence and confocal microscopy analysis

Immunostaining and CLSM analyses were carried out following standard techniques, using the appropriate primary antibodies (Table 2) and the corresponding secondary fluorochrome‐conjugated antibodies (Jackson ImmunoResearch Laboratories). Images were acquired with the confocal laser system TCS‐SP5, Leica. Quantification of nucleus/cytoplasm fluorescence intensity was performed as reported previously (Aguilar et al., 2015). Morphometric analysis of nuclei was performed with software ImageJ 1.46j. Nucleolar area was quantified by measuring the cross‐sectional area occupied by a mark corresponding to fibrillarin stain, with software ImageJ 1.46j.

**Table 2:** List of antibodies and plasmids used in this study

Antibodies				
Antibody target	Supplier	Host	Dilution	
			WB	IF
STAT3	Santa Cruz Biotechnology (Cat: sc−482)	Rabbit polyclonal	1:3,000	1:50
B23	Santa Cruz Biotechnology (Cat: sc−6013‐a)	Rabbit polyclonal	1:3,000	1:50
Cyclin B1	Santa Cruz Biotechnology (Cat: sc−752)	Rabbit polyclonal	N/A	1:100
GFP	Santa Cruz Biotechnology (Cat: sc−8334)	Rabbit polyclonal	1:1,500	N/A
CRM1	Novus Biologicals, (Cat: NB100−79802)	Rabbit polyclonal	1:4,000	1:250
Lamin B1	Abcam (Cat: ab16048)	Rabbit polyclonal	1:3,000	1:250
Fibrillarin	Abcam (Cat: ab5821)	Rabbit polyclonal	N/A	1:50
H3K9me3	Abcam (Cat: ab8898)	Rabbit polyclonal	1:3,000	1:1,500
ZO−2	Zymed Laboratories (Cat: 71–1400)	Rabbit polyclonal	1:1,000	1:200
FLAG	Cell Signaling Technology (Cat: 2368)	Rabbit polyclonal	1:1,000	N/A
Dp71	Genemed Synthesis (Cat: +78Dp71)	Rabbit polyclonal	N/A	1:100
Lamin A/C	Santa Cruz Biotechnology (Cat: sc−20681)	Rabbit polyclonal	1:4,000	N/A
Lamin A/C	Hybridoma Bank (Cat: MANLAC3(4C10))	Mouse monoclonal	N/A	1:10
HDAC	Santa Cruz Biotechnology (Cat: sc−7872)	Rabbit polyclonal	N/A	1:60
NTF2	Santa Cruz Biotechnology (Cat: sc−80008)	Mouse monoclonal	1:150	1:50
Phospho‐p70 S6K	Santa Cruz Biotechnology (Cat: Sc230)	Rabbit polyclonal	1:300	N/A
Phospho‐rpS6	Cell Signaling Technology (Cat: 4857)	Rabbit monoclonal	1:1,000	N/A
Phospho‐eIF2α	Cell Signaling Technology (Cat: 9721)	Rabbit polyclonal	1:1,000	N/A
Actin	Dr. Manuel Hernández, CINVESTAV, Mexico	Mouse monoclonal	1:3,000	N/A
NF‐YA	Santa Cruz Biotechnology (Cat: sc−17753 X)	Mouse monoclonal	N/A	N/A

Plasmids	
Name	Supplier
pBABE‐puro‐GFP‐wt‐lamin A	Addgene
pBABE‐puro‐GFP‐progerin	Addgene
pFLAG‐hCRM1	Addgene
p3xFLAG‐CMV−10	Addgene
Flag‐CRM1	This study
pSG5‐LB1	MSc Paulina Azuara, CINVESTAV, Mexico
pEGFP‐C1	Addgene
pEGFP‐C1‐LB1	This study
CSHCTR001‐nH1	GeneCopoeia
CS‐HSH090209‐nH1−01a	GeneCopoeia
pCRM1promoter	This study

### 4.5 Senescence‐Associated β‐Galactosidase (SA‐β‐Gal) Assay

Fibroblasts seeded on coverslips were stained with SA‐β‐Gal following manufacturer's instructions (Senescent Cell Histochemical Staining Kit, Sigma‐Aldrich). Blue‐stained cells expressing β‐galactosidase (senescent cells) were observed under bright‐field microscopy.

### 4.6 Western blotting

Immunoblotting analyses were carried out following standard protocol. Specific proteins were visualized using the Enhanced Chemiluminescence (ECL™) Western blotting detection system (Amersham Pharmacia, GE Healthcare), according to the manufacturer's instructions.

### 4.7 Flow cytometry and cell proliferation assays

The analysis of cell cycle was carried out on a BD LSRFortessa flow cytometer (BD Biosciences), using the ModFit LT software (Verity Software House). Cell proliferation was assessed by the 3‐(4,5‐dimethylthiazole‐2–5‐diphenyl tetrazolium bromide (MTT) assay, as reported previously (Velez‐Aguilera et al., 2018).

### 4.8 Quantitative reverse transcription PCR (RT‐qPCR)

Total RNA was isolated from fibroblasts using the Direct‐zol^TM^ RNA MiniPrep Kit (Zymo Research), according to the manufacturer's instructions. RT‐qPCR was carried on the StepOnePlus Real‐Time PCR System (Applied Biosystems), using the KAPA SYBR Fast One‐Step qRT‐PCR system (Kapa Biosystems) and following a standard protocol. The expression levels for *CRM1* mRNA were measured by the 2^ΔΔct^ method and normalized to *GAPDH* mRNA values. Primer sequences for CRM1 and GAPDH amplification will be provided under request.

### 4.9 Statistical analysis

Statistical analyses were performed by the two‐tailed unpaired Student's *t* test. Data represent the mean ± SEM from a series of three separate experiments, and *p* values < 0.05 are indicative of statistical significance. Where indicated, statistical analysis was carried out using exact nonparametric Mann–Whitney U test or unpaired *t* test and data were summarized by the mean ± SEM or the mean ± *SD* from a series of three separate experiments. *p *values < 0.05 were considered as significant. All statistical analyses were performed using GraphPad Prism 5 software (San Diego).

## CONFLICT OF INTEREST

None declared.

## **AUTHOR** **CONTRIBUTIONS**

IGA and AAI conceived and designed the study; acquired, analyzed, and interpreted the data; and drafted/revised the article. RP designed the study, and acquired and analyzed the data. GVA and AE participated in investigation, contributed to methodology, and acquired and interpreted the data. GEJG and AVL acquired the data. MSLC conceived and designed the study. JM reviewed the paper and collected the resources. SJW designed, wrote, reviewed, and edited the article. BC conceived and designed the study, collected the resources, performed formal analysis, supervised the study, acquired funding, wrote the original draft, administered the project, and reviewed and edited the article.
