# Supplementary material for: Enhanced nuclear protein export in premature aging and rescue of the progeria phenotype by modulation of CRM1 activity
Source: Aging Cell. 2019 Jul 15;18(5):e13002. doi: 10.1111/acel.13002 (PMC6718587; doi:10.1111/acel.13002)

Supplemental Figure 1

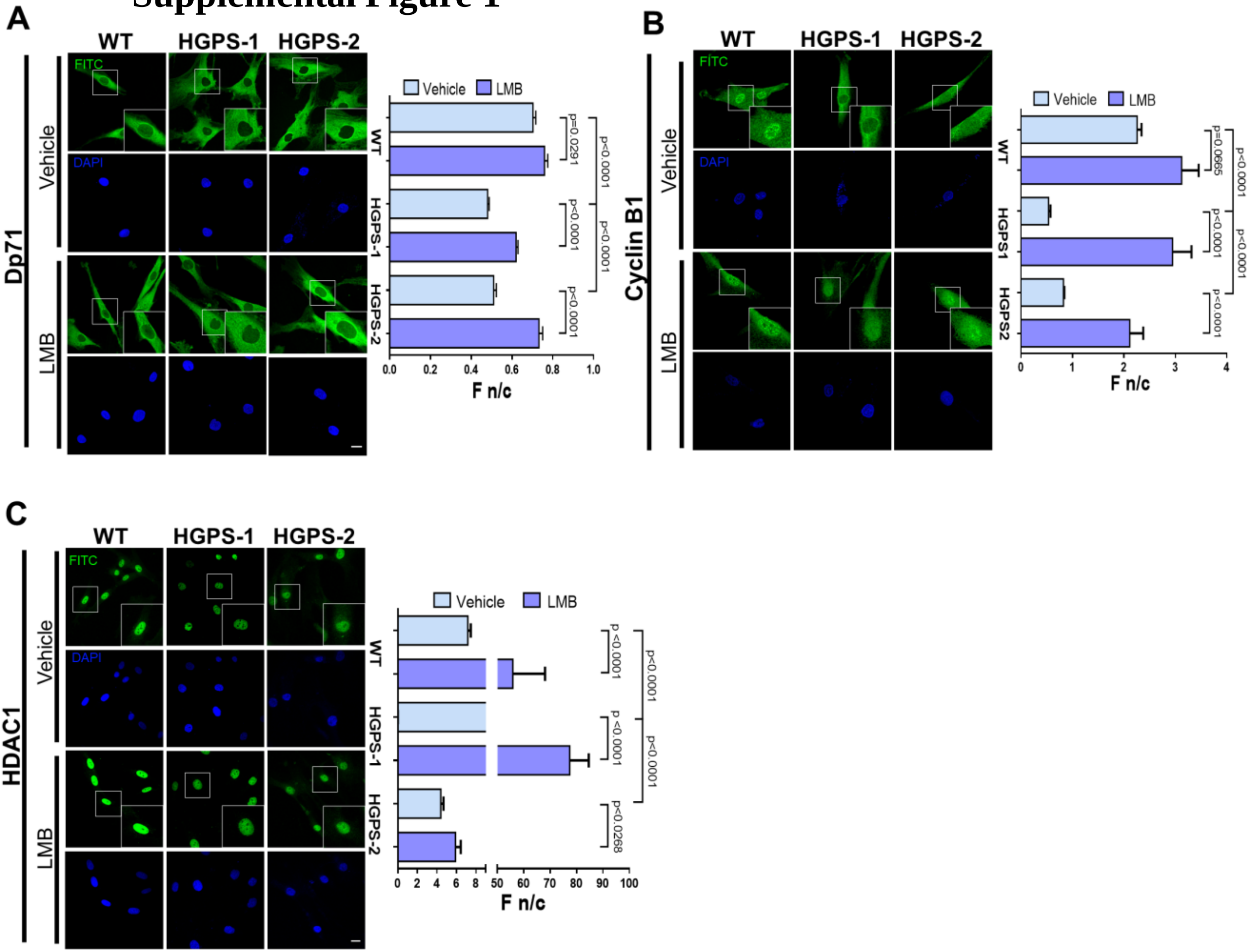

Supplemental Figure 2

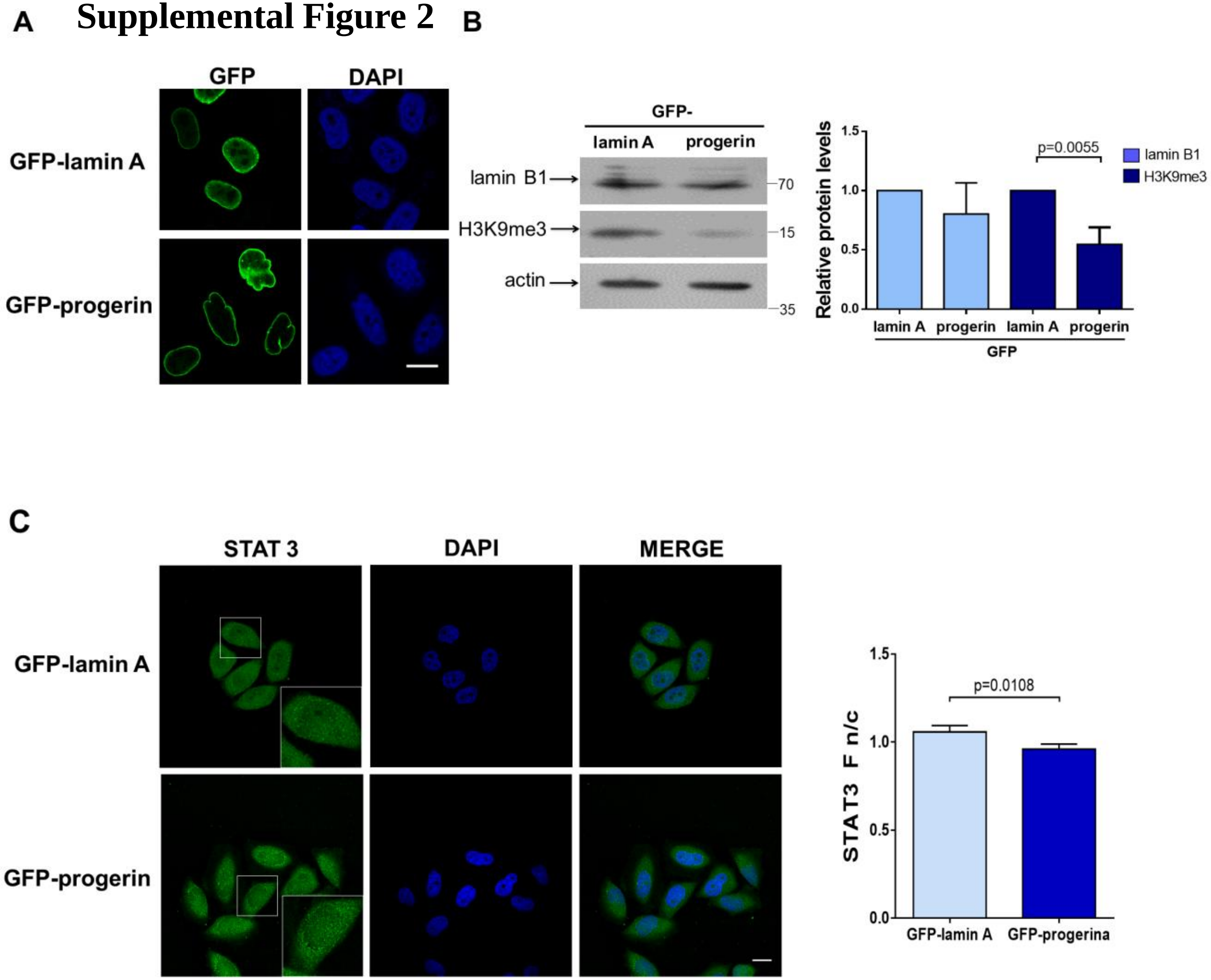

Supplemental Figure 3

A

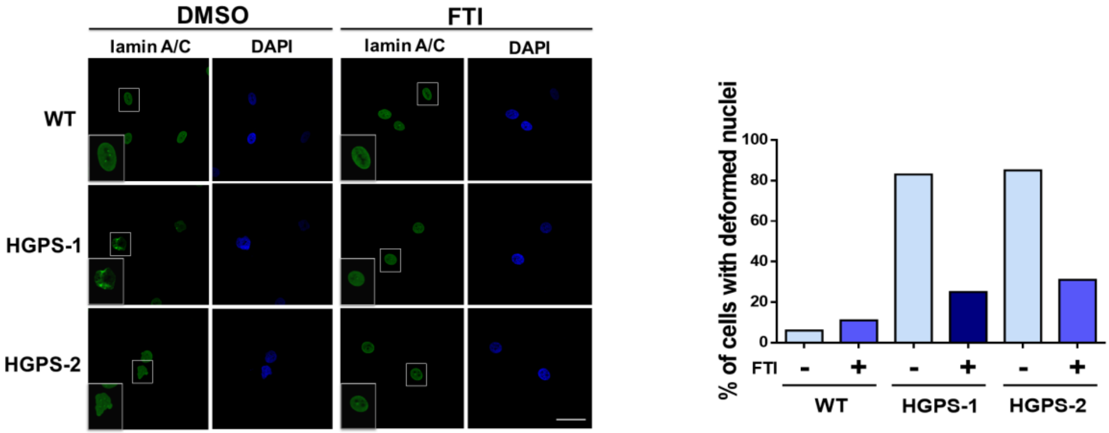

B

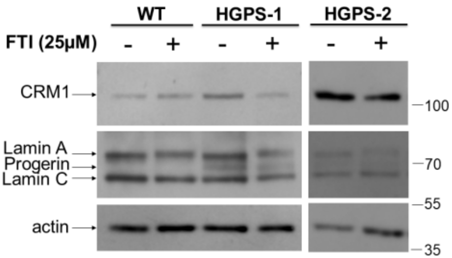

C

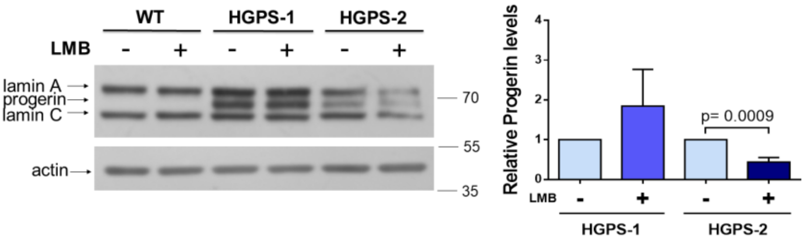

Supplemental Figure 4

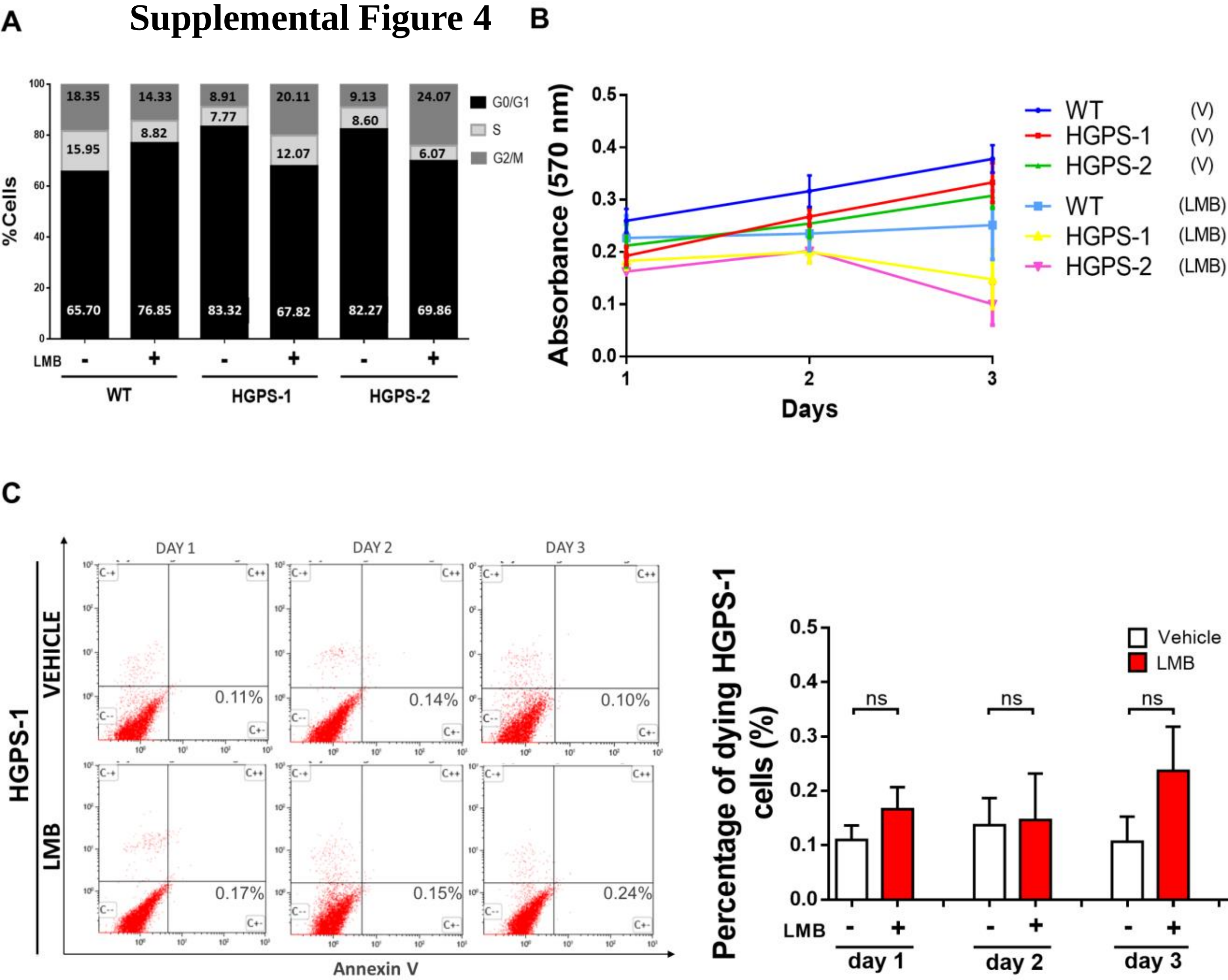

Supplement: Supplementary file 1 [file ACEL-18-e13002-s001.pdf]
